# Domestic Correspondence

**Published:** 1890-03

**Authors:** 


### Domestic Correspondence.

To the Editor:

*First District Dental Society, State of New York.*—The Twenty-first Anniversary of this Society took place in New York City during the third week in January. Clinics were given in the rooms of the New York College of Dentistry on Tuesday, Wednesday, and Thursday, and a large number of dentists performed operations on each day of the session, which were witnessed by crowds of interested spectators. Meetings of the Society were also held in the spacious hall of the Masonic Temple, where a number of interesting papers were read and ably discussed, and where various specimens were exhibited to aid in demonstrating theories advanced by some of the speakers.

According to reports in the New York daily papers, several hundred dentists were in attendance. Among the guests present were Dr. J. S. Campbell, of London, Eng.; Dr. J. H. McKellops, of St. Louis; Drs. J. L. Gish and C. M. Chase, of Michigan; Drs. M. W. Foster, T. S. Waters, and W. Finney, of Baltimore; Dr. Geo. H. Wells, of Augusta, Ga.; Drs. E. S. Talbot, J. N. Crouse, A. W. Harlan, and W. B. Ames, of Chicago; Drs. L. D. Shepard, H. K. Stoddard, J. E. Waitt, and F. M. Dowsley, of Boston; Drs. W. C. Barrett and F. E. Howard, of Buffalo; Drs. Jas. Truman, C. N. Peirce, and L. A. Faught, of Philadelphia; Drs. S. B. Palmer and G. L. Curtis, of Syracuse; Drs. E. C. Baxter, of Albany; F. B. Darby, of Elmira; Fred. H. Lee, of Auburn, N. Y.; Dr. E. V. McLeod, of New Bedford, Mass.; Drs. C. S. Stockton, J. E. Palmer, G. E. Adams, F. A. Levy, A. R. Eaton, Oscar Adelberg, B. F. Lucky, C. A. Meeker, C. S. G. Watkins, F. C. Barlow, C. F. Holbrook, and W. Pinney, all of New Jersey; Drs. E. S. Gaylord and Jos. H. Smith, of New Haven, Conn.; Drs. O. E. Hill and N. Jarvie, of Brooklyn, and Dr. Carl Heitzmann, of New York. Many other visiting dentists were present whose names could not be secured for this report.

A dinner was given by the Society to the invited guests on Thursday evening at Clark's famous Café, in Twenty-third Street, near Fifth Avenue. Unfortunately many of the visitors had left the city for their homes, as is apt to be the case where the banquet is deferred until the last evening of a session; however, over one hundred gentlemen occupied chairs at the tables. The dinner was becoming the occasion and was well served. Everybody present looked happy and all were apparently engaged in cheerful conversation, yet doing good service in disposing of the tempting viands placed before them.

The Brooklyn and Jersey delegations were particularly jovial, as is the usual way with them on such occasions. Hill was in his best mood; Stockton was aglow with wit and humor; Meeker became decidedly hilarious; Levy was all radiant with smiles; Pinney was almost boisterous, and even Adams, who is usually so meek and gentle, looked as though he felt somewhat the influence of the "spirit" of the hour.

Chicago was not much behind them in enthusiasm. There was Crouse, the inevitable, who cannot be crushed,—the indefatigable worker and champion of his profession, who is bound to have his fellow-dentists protected from unjust extortions of wolves and sharks in human attire. Harlan occupied a position just opposite, drinking it all in and rolling up ideas which may appear in a future number of the *Review*. Beyond him sat Talbot, who has the reputation of being exceedingly regular in all his habits and individual ways, but who is ever on the alert to pry into and study up "irregularities" in others. Foster, of Baltimore, secured a seat at the president's elbow, *presumably* in order to be fully served, and evidently succeeded. McKellops, of St. Louis, known everywhere as "Mac," was "around," and held his own in his accustomed genial way.

In due time, the order of "menu" being completed, President Northrop sounded the signal for order, and, after a brief address, introduced Dr. Wm. Jarvie, of Brooklyn, to respond to the toast, "The Dental Profession." Dr. Jarvie spoke of the present advanced condition of dentistry; also of the successful professional career of prominent dentists in this country and Europe, and of the high social position they had attained. In referring to examinations of students, or applicants for diplomas, he stated that, in his opinion, "true merit and ability," if satisfactorily demonstrated, should have greater weight in the deliberations of the examining board than the length of time spent in a dental college.

Dr. A. W. Harlan, of Chicago, was called upon to respond to the sentiment offered for "Our Guests." The doctor believed that the guests had greatly enjoyed the hospitality of their New York friends, and had been much interested in the meetings of the society.

To the next toast, "The First District Dental Society," Dr. N. W. Kingsley was called upon to speak. In the order of travesty, Dr. Kingsley gave an amusing account of the life and workings of the society from the time of its organization, or "birth," to the present period of its maturescent loveliness. The audience was kept in a state of continuous laughter throughout the narrative.

To the fourth toast, "The Jersey Boys," Dr. C. S. Stockton, of Newark, was requested to respond. The doctor spoke well for the "boys," and thinks them equal to any occasion like the present one. They, too, know how to get up excellent meetings and how to entertain their visiting friends. He hopes to welcome them all in Jersey in 1892, if the World's Fair Committee decide to have the fair held on Jersey territory. The doctor complimented the New York society on the success of its recent meetings, and the splendid entertainment which supplemented it.

When the last toast, "The Dental Protective Association of the United States," was announced, Dr. J. H. Crouse, of Chicago, was loaded for a response which he fired off with telling effect. He referred to the immense amount of work done by the officers of the Protective Association without fee or reward, and asked of each dentist only the small sum of ten dollars to make up a fund sufficient to protect them in the practice of their specialty and to defend them from unjust exactions.

Mr. J. Kimberly Beach, of New Haven, Conn., was called upon for a speech. He is a member of the legal profession, and envies the dentists for the high position they have gained. He complimented them for the many achievements they had won, and believes that in the great future before them dentistry will be generally recognized as a most useful, benign, and respected specialty of medicine. He thinks that dentists should be protected from unjust claims of pretenders, and in order to be protected should support the association.

Dr. W. H. Dwinelle made a few remarks complimentary to the efforts of Dr. Crouse; after which Dr. C. E. Francis called the attention of the gentlemen present to the arduous labors of Dr. Crouse, and to the valuable time gratuitously given by him for the benefit of dentists generally. Dr. Francis also offered a resolution of thanks to Dr. Crouse for his efforts in behalf of his specialty, which by vote was unanimously carried.

After a song by Dr. B. C. Nash, in which the entire assembly heartily joined, the happy party adjourned.

